# Correlates of Vaginal Colonization with Group B Streptococci among Pregnant Women

**DOI:** 10.4103/0974-777X.68536

**Published:** 2010

**Authors:** Tsering Chomu Dechen, Kar Sumit, Pal Ranabir

**Affiliations:** *Department of Microbiology, Sikkim Manipal Institute of Medical Sciences (SMIMS) and Central Referral Hospital (CRH), 5^th^ Mile, Tadong, Gangtok, Sikkim-737102, India*; 1 *Department of Community Medicine, Sikkim Manipal Institute of Medical Sciences (SMIMS) and Central Referral Hospital (CRH), 5^th^ Mile, Tadong, Gangtok, Sikkim-737102, India*

**Keywords:** Group B streptococcus, Pregnancy, Women

## Abstract

**Background:**

A study of genital colonization by group B streptococcus (GBS) was conducted in pregnant women in their third trimester, which is a known risk factor of morbidity and mortality among newborns.

**Aims:**

The present study was undertaken to study the prevalence and the correlates of vaginal colonization by GBS among pregnant women.

**Setting and Design:**

This observational cross-sectional study was conducted during September 2002 to March 2004 on 524 pregnant women.

**Materials and Methods:**

Three high vaginal swabs were obtained from all the pregnant women admitted at term and in preterm labor. Two swabs were used for aerobic culture and the third one for gram staining. The first set of swabs was cultured on 5% Sheep blood agar plates. The second set of swabs were inoculated into Todd–Hewitt broth and then subcultured in 5% Sheep blood agar plates. The main outcome measures were the presence of GBS infection in comparison to the age group, gravida, gestational age, premature rupture of membrane (PROM), preterm labor and association with febrile spells of the present pregnancy.

**Results:**

The culture positivity rate of GBS was 4.77% and coexistent organisms isolated were *Candida species* (36%), *Staphylococcus aureus* (8%) and *Enterococcus species* (8%). Culture positivity in the age group of 18–25 years was 5.71%, of which 5.74% were in their first pregnancy. The correlation between age group and gravida with GBS culture positivity was statistically insignificant. The culture positivity in <36 weeks of gestational age was 6.93%. This relation was statistically significant. Twenty-eight percent developed PROM. Sixty-four percent of culture positives had preterm labor.

**Conclusion:**

GBS infection among pregnant women was significantly correlated with the gestational age, PROM and preterm labor. In pregnancy GBS colonization causes asymptomatic bacteriuria or UTI. It is a well known cause of puerperal infections with amnionitis, endometritis and sepsis being the most commonly reported infections.[5]

## INTRODUCTION

Group B streptococci (GBS) are a constituent of the normal vaginal bacterial microflora, which often do not demonstrate any clinical symptoms. On the other hand, during pregnancy, there are optimal conditions for GBS multiplication in the vagina, which may have very serious consequences for both the mother and her child. The researchers in this field in the mid-1980s demonstrated that GBS was carried in the vaginal and anorectal flora of up to 30% of women, which can be intermittent, transient or persistent.[1,2]

GBS continue to be major perinatal pathogens, both for mothers and their infants, and are associated with significant morbidity and mortality, and their attendant cost to society as life-threatening emergency and any delay in treatment may cause death. Early diagnosis and proper management of this preventable menace of vaginal colonization with GBS among pregnant womencan bring down the morbidity and mortality substantially. Perinatal infections are one of the fundamental causes of early puerperal complications in mothers and neonates. While different preventive strategies to identify women at risk are being recommended, the optimal strategy depends on the incidence of GBS-sepsis and on the prevalence of anogenital GBS colonization.[3,4]

In pregnancy GBS colonization causes asymptomatic bacteriuria or UTI. It is a well known cause of puerperal infections with amnionitis, endometritis and sepsis being the most commonly reported infections.[5]

Pregnant women who are GBS carriers have the potential to transmit the organism to their newborn infants. There is a spectrum of maternal and fetal GBS infections ranging from asymptomatic colonization to sepsis.

GBS has been implicated in adverse pregnancy outcomes, including premature rupture of membranes (PROM), preterm labor and clinical and subclinical chorioamnionitis.[6]

GBS is a leading cause of morbidity and mortality among newborns. Universal screening for GBS among women at 35–37 weeks of gestation is more effective than administration of intrapartum antibiotics based on risk factors. Studies indicate that intrapartum prophylaxis of GBS carriers and selective administration of antibiotics to newborns reduce neonatal GBS sepsis by as much as 80–95%.[7]

Women with GBS colonization are at an increased risk of GBS colonization in a subsequent pregnancy. Prior GBS colonization should be considered in the algorithm to treat unknown GBS status during term labor.[8]

Investigators tried to find out/identify risk factors that may influence the prevalence of GBS, like ethnicity, smoking, maternal age and number of partners. The colonization rates are incoherent enough to target only high-risk women and may not be an effective strategy.[9,10]

Pregnant women who are GBS carriers have the potential to transmit the organism to their newborn infants. There is a spectrum of maternal and fetal GBS infections ranging from asymptomatic colonization to sepsis.

GBS has been implicated in adverse pregnancy outcomes, including premature ROM, preterm labor and clinical and subclinical chorioamnionitis.[10] The Centers for Disease Control (CDC) call for antibiotic prophylaxis in women with asymptomatic first trimester bacteriuria because this is ‘a marker for heavy genital tract colonization,’ and screening all other women at 35–37 weeks for vaginal and rectal colonization.[11]

Hence, the present study was undertaken to study the bacteriological profile of GBS among pregnant women and their antimicrobial sensitivity pattern for planning strategy for the management of these cases. To the best of our knowledge, this study was one among the few researches conducted in India on variables of GBS vaginal colonization among pregnant women.

## MATERIALS AND METHODS

### Study period

September 2002 to March 2004.

### Study population

The study included 524 pregnant women admitted at term and in preterm labour. The pregnant women who were on antibiotic intervention in the last trimester of pregnancy were however excluded from the study population. The main outcome measures were presence of culture-positive GBS infection in comparison to the age group of the mothers, parity and gestational age of the present pregnancy.

### Data collection procedure

The study was conducted in Lady Goschen Hospital during September 2002 to March 2004 on 524 pregnant women. Institutional ethics committee approved the study. All the subjects were explained about the purpose of the study and were ensured strict confidentiality. Written informed consents were taken from each of the caregivers of the children before the study. Following Helsinki Declaration on research bioethics, the participants and their caregivers were given the option not to participate in the study if they wanted otherwise. Standard microbiological methods were followed in this study during swab culture and antibiotic sensitivity test. Specimens were collected with the help of serum-coated cotton-tipped swab sticks for culture following universal precautions. Three high vaginal swabs were obtained. Two swabs were used for aerobic culture (one for streaking solid media and the other for inoculation into liquid media) and the third was for gram staining.

The swabs were inoculated into Stuart’s transport media in the test tubes and processed within 30 min. The first set of swabs were cultured on 5% Sheep blood agar plate and incubated at 37° C in a candle jar containing 10% CO_2_ for a period of 18–24 h. The agar surface was examined for β-hemolytic colonies.[12,13]

The second set of swabs were inoculated into Todd–Hewitt broth and incubated at 37° C for 18–24 h. After 24 h, the broth was observed for turbidity and then it was subcultured onto 5% Sheep blood agar plates. On 5% Sheep blood agar, GBS produce a relatively narrow zone of β-hemolysis.[14]

### Statistical analysis using microsoft excel

The data collected were thoroughly cleaned and entered into Microsoft Excel spread sheets and analysis was carried out. The procedures involved were transcription, preliminary data inspection, content analysis and interpretation. Percentages were used in this study to analyze epidemiological variables.

## RESULTS

In this study, vaginal swabs were obtained from all the 524 pregnant women admitted at term and in preterm labor in our tertiary care center. Twenty-five of them were observed to be culture positive for *Streptococcus agalactiae*, GBS, with a culture positivity rate of 4.77%. Culture positivity among the mothers in the age group of 18–25 years was 5.71%, followed by the age group 26–30 years. Of all the primi mothers under study, 5.74% of culture-positive mothers were in their first pregnancy. The interesting observation was that the infection rate decreased as the gravida of the mother increased. These correlations between age group of mothers and gravida with GBS culture positivity was however statistically nonsignificant. The culture positivity among the mothers <36 weeks in gestational age was 6.93%, while that among mothers >36 weeks was far less. This difference was statistically significant [Table 1]

**Table 1:** Prevalence and correlates of vaginal colonization with GBS among pregnant women

	No. of total cases	No. of cases colonized for GBS	Percentage	Statistical significance
Age group of mothers				
18–25 years	210	12	5.71	X2 = 0.85; P>0.05
26–30 years	170	8	4.70	
31–35 years	93	3	3.22	
>35 years	51	2	3.92	
Total	524	25	4.77	
Parity of the mother				
Primi	244	14	5.74	X2 = 0.73; P>0.05
G-2	192	8	4.17	
G-3	57	2	3.50	
>=G-4	31	1	3.23	
Total	500	25	4.77	
Gestational age of mothers				
<36 weeks	231	16	6.93	X2 = 4.23; P<0.05
>36 weeks	293	9	3.07	
Total	524	25	4.77	

The spectrum of organisms isolated included GBS, *Candida species, Staphylococcus aureus and Enterococcus species*. The coexistent organisms isolated from the culturepositive cases were *Candida species* (36%), *Staphylococcus aureus* (8%) and *Enterococcus species* (8%) [Table 2].

**Table 2:** Microorganism coinfection with GBS

Organism	No. among positive-culture cases	Percentage
Candida spp	9	36
Staphylococcus aureus	2	8
Enterococcus spp	2	8

The majority of such cases (5.74%) were the primi mothers under study. Culture positivity among the mothers in the age group of 18–25 years was 5.71%, followed by the age group 26–30 years. Seven out of 25 (28%) of the mothers found to be culture positive for GBS developed premature ROM and draining of amniotic fluid. Sixty-four percent (64%) of GBS culture-positive mothers had preterm labor. Among the 25 women who were colonized with GBS, five developed fever (20%). Of these five cases, one case developed fever antepartum and four postpartum [Table 3].

**Table 3:** Incidence of complication following GBS genitourinary tract colonization during pregnancy

Complications	No. of cases colonized for GBS	No. of cases with complications	Percentage
Incidence of PROM	25	7	28
Incidence of preterm labor	25	16	64
Fever during late pregnancy	25	5	20

## DISCUSSIONS

The culture positivity rate of GBS was 4.77% and coexistent organisms isolated were *Candida species* (36%), *Staphylococcus aureus*, (8%) and *Enterococcus species* (8%). Culture positivity in the age group of 18–25 years was 5.71%, of which 5.74% were in their first pregnancy. The correlation between age group and gravida with GBS culture positivity was statistically insignificant. The culture positivity in <36 weeks of gestational age was 6.93%, which was statistically significant. Twenty-eight percent developed premature ROM. Sixty-four percent of culture positives had preterm labour. Of the febrile cases, a single case developed fever antepartum and four postpartum.

Researchers from Poland in a study with 563 women in their third trimester either in normal or high-risk pregnancy opined that GBS colonization was 20% in high-risk pregnancy and, in normal pregnancy, it was found to be 17.2%. Both in the high-risk group and in the newborns, we confirmed a higher and statistically significant frequency of detection of GBS strains.[5]

In a study in Saudi Arabia, the GBS colonization rate among term Saudi pregnant women is relatively high (27.6%) and rectal swab specimens at late pregnancy appeared necessary to accurately identify GBS maternal colonization.[15]

A study from Trinidad conducted on 204 pregnant women in their third trimester reflects that 64 (31.4%) of these women had positive GBS culture from vaginal and rectal swabs. No significant differences in colonization rates were noted on the basis of ethnicity (race) and gravidity. There was a significant trend of increasing prevalence with increasing age. Colonization was not significantly greater in multigravida than in primigravida women. There was no significant differences between colonization in Negro (black) women and colonization in East Indian women.[16]

In a study from the United States, GBS was detected in 13.31% of patient self-collected samples and 10.65% of physician-collected specimens (relative risk, 1.25; 95% CI, 0.85–1.84). The study had 90% power to detect a 10% difference in colonization rates.[17]

In a study from Brazil to assess the prevalence of GBS colonization, GBS was 20.4% in pregnant women in labor of a public maternity center. There was no association between the sociodemographic variables or gynecological–obstetrical antecedents, and a larger presence of GBS colonization. The prevalence of GBS colonization was high among the mothers, similar to that described in other studies.[18]

In a study performed in Iran to evaluate the prevalence of rectovaginal colonization with GBS among pregnant women, of the 1,197 pregnant women who were evaluated for GBS, 110 (9.1%) had rectovaginal colonization. The mean gestational age of newborns in this group was 32.8+/−11 weeks. In this study, 9.1% of the women had positive rectovaginal GBS, cultures with a 60% transmission rate to their neonates. Also, preterm birth, prolonged ROM and preterm premature ROM had a higher incidence among GBS-colonized mothers.[19]

A study was conducted in the obstetric and neonatal wards to assess the actual rates of colonization of pregnant women and their children with GBS in a Polish University Hospital. Resistance of these cocci to macrolides and clindamycin was also tested and the routes of transmission of GBS were followed in some cases using molecular typing. Colonization with GBS was checked in 340 pregnant women living in the south-eastern region of Poland (Małopolska) in the years 2004–2006. Women with a complicated pregnancy were more often colonized than those with a normal pregnancy (20.0% versus 17.2%). Moreover, women with a complicated pregnancy were twice as often colonized with GBS strains with the MLS(B) phenotype. Our results clearly indicate that rates of GBS colonization among pregnant women and neonates in a Polish University Hospital have reached levels comparable to those reported in other European clinical centers.[20]

A study was conducted in the Netherlands to determine the relationship between maternal colonization with GBS and preterm delivery. The researchers reported that the combined estimate from a random effect metaanalysis of the 11 cohort studies was 1.06 (95% CI, 0.95–1.19) and for the five cross-sectional studies was 1.75 (95% CI, 1.43–2.14). For the case control studies, the pooled odds ratio was 1.59 (95% CI, 1.03–2.44). This systematic review did not show an association between maternal GBS colonization during pregnancy and preterm delivery. However, in case of preterm delivery, there is an increased risk of subsequent maternal GBS colonization.[21]

In another study conducted in Thailand to determine the risk factors related to GBS colonization in pregnant women, 320 pregnant women, who fulfilled the specified criteria, were selected for a cross-sectional descriptive study. Swabs were cultured from the lower vagina and anorectum for GBS using Todd–Hewitt broth, like in our study. Colonization was present in 58 cases (18.12%). The risk factor for GBS colonization was an older mean maternal age and a lower mean gestational age. No mothers or neonates during the study period developed a clinical infection from GBS. The risk factors for GBS colonization in pregnant women were older maternal age and lower gestational age.[22]

In a retrospective cohort study conducted in the USA on women who had two consecutive deliveries with the availability of GBS culture result at 35–37 weeks of gestation, or the diagnosis of GBS colonization to estimate the prevalence of GBS colonization in a subsequent pregnancy in women with and without GBS colonization in an index pregnancy, a total of 102 women positive for GBS genitourinary colonization were compared with controls. The rate of recurrence for GBS colonization (53%) was significantly higher when judged against women who were GBS-negative in their index pregnancy (15%) (adjusted odds ratio, 11.7; 95% CI, 3.5–38.9; *P*<0.01). Women who were GBS positive in the index pregnancy were more often of African-American race and less likely to be nulliparous or smoke tobacco. Women with GBS colonization are at an increased risk of GBS colonization in a subsequent pregnancy. Prior GBS colonization should be considered in the algorithm to treat unknown GBS status during term labor.[8]

### Strengths of the study

The study’s strength lies in dealing with the novel idea. It was a significant and important issue. We presented an analysis of GBS disease in a tertiary healthcare center to analyze the factors that will directly have an impact on reducing morbidity and thus influencing the overall medical care available for mothers. The percentages were apparent only among all the pregnant women within this referral center during the study period.

The strength of the study also lies in finding diverse patterns of microbiological behavior and considerable variation in geographical distribution from other studies in India.

### Limitations of the study

This study contained new and unique idea that evaluated correlations between overall GBS positivity and subject demographics that may influence various emergency conditions. The study could not be followed-up later to understand the morbidity profile in the neonates.

### Future directions of the study

Research on GBS had been performed globally for decades to understand better maternal care. The need for screening all the pregnant mothers for GBS colonization was growing day by day as a result of advancement in medicine. Considering the accelerated demand for reducing neonatal morbidity and mortality due to GBS, it was surprising that so little research had been performed in this field in India. In most of the studies conducted in this field, it was found that early diagnosis and proper management of GBS among pregnant women can bring down the morbidity and mortality due to neonatal septicemia substantially. There is an urgent need to define the role of community-based management. It should be routine in a tertiary care center and it should be stored in a dynamic database. We need prospective population-based studies to elucidate the national incidence and to identify the risk factors for GBS among pregnant women. Investigations were also needed to assess research directions to develop well-designed interventions. Modern sources of positive and negative impetus were worth exploring through scientifically sound studies using multipronged approaches. Investigations were also needed also to assess research questions and to develop well-designed interventions to test hypotheses and to produce generalizable future observations.

## CONCLUSION

The finding of the research observed the spectrum of microbial pathogens in relation to GBS among pregnant women. GBS continue to be major perinatal pathogens, both for mothers and their infants, and are associated with significant morbidity, mortality and its attendant cost to society. Approaches to prevention are directed toward either eliminating exposure to the organism or enhancing host resistance, i.e., chemoprophylaxis and immunoprophylaxis. Intrapartum chemoprophylaxis has been shown to effectively interrupt vertical transmission of GBS from the genitally colonized mother to the infant and to decrease the incidence of both maternal and early-onset neonatal GBS disease. To avoid unnecessarily exposing large numbers of colonized women to antibiotics, only those with defined risk factors should be selected for intrapartum chemoprophylaxis. Immunoprophylaxis and active immunization, in particular, are the most promising methods of preventing perinatal GBS disease in mothers and their infants, including late-onset disease. Immunization of pregnant women with type III polysaccharide vaccine has resulted in adequate provision of functional antibody to the infants born to responders.[23]
